# Ferroptosis: a new antidepressant pharmacological mechanism

**DOI:** 10.3389/fphar.2023.1339057

**Published:** 2024-01-08

**Authors:** Guangheng Zhang, Shimeng Lv, Xia Zhong, Xiangyu Li, Yunhao Yi, Yitong Lu, Wei Yan, Jiamin Li, Jing Teng

**Affiliations:** ^1^ Department of First Clinical Medical College, Shandong University of Traditional Chinese Medicine, Jinan, China; ^2^ Wangjing Hospital, China Academy of Chinese Medical Sciences, Beijing, China; ^3^ College of Traditional Chinese Medicine, Shandong University of Traditional Chinese Medicine, Jinan, China; ^4^ Affiliated Hospital of Shandong University of Traditional Chinese Medicine, Jinan, China

**Keywords:** ferroptosis, depression, traditional Chinese medicine, pharmacological mechanism, antidepressants

## Abstract

The incidence rate of depression, a mental disorder, is steadily increasing and has the potential to become a major global disability factor. Given the complex pathological mechanisms involved in depression, the use of conventional antidepressants may lead to severe complications due to their side effects. Hence, there is a critical need to explore the development of novel antidepressants. Ferroptosis, a newly recognized form of cell death, has been found to be closely linked to the onset of depression. Several studies have indicated that certain active ingredients can ameliorate depression by modulating the ferroptosis signaling pathway. Notably, traditional Chinese medicine (TCM) active ingredients and TCM prescriptions have demonstrated promising antidepressant effects in previous investigations owing to their unique advantages in antidepressant therapy. Building upon these findings, our objective was to review recent relevant research and provide new insights and directions for the development and application of innovative antidepressant strategies.

## 1 Introduction

Major depressive disorder (MDD), commonly known as depression, is a prevalent mental disorder that poses a significant threat to both physical and mental wellbeing. Its primary clinical manifestations include persistent emotional depression, reduced interest, intellectual disability, cognitive impairment, sleep disturbances, and other psychiatric symptoms. In severe cases, it can even lead to suicidal tendencies (JIAO et al., 2021). The World Health Organization (WHO) predicts that by 2030, depression will be the leading cause of disability worldwide (BARNETT, 2019). Currently, selective serotonin reuptake inhibitors (SSRIs) and other medications are primarily used for treatment. SSRIs selectively inhibit serotonin (5-HT) transporters and block the reuptake of 5-HT, thereby enhancing its effect and producing antidepressant effects (KRAUS et al., 2017). However, these treatments are often associated with adverse reactions such as nausea, headaches, sexual dysfunction, and weight gain. Furthermore, they exhibit delayed efficacy and high non-response rates (WU S. et al., 2021). Consequently, there is an urgent need to develop more effective and safer antidepressant treatments. Traditional Chinese medicine (TCM) possesses characteristics such as multi-component, multi-target, and multi-channel effects, making it a promising approach for depression treatment. Some active ingredients derived from TCM have demonstrated significant antidepressant efficacy without notable toxic side effects (CHI et al., 2019), providing extensive research opportunities in the field of antidepressant therapy.

Despite advancements, the precise pathological mechanism underlying depression remains incompletely understood (DEAN and KESHAVAN, 2017). Ferroptosis, a newly discovered form of programmed cell death first proposed in 2012, has gained considerable attention in recent years. Iron, as the most abundant transition metal element in the brain, plays a crucial role in various physiological processes, including myelin formation, neurotransmitter synthesis and transmission, and oxidative metabolism of nerve cells (LEI et al., 2021). Consequently, the brain is highly vulnerable to alterations in iron homeostasis (FERREIRA et al., 2019). Disruptions in iron homeostasis can lead to neuronal damage (MAHER, 2018), which is closely associated with the onset of various neurodegenerative diseases, such as Parkinson’s and Alzheimer’s disease (MAHONEY-SÁNCHEZ et al., 2021). Emerging studies have indicated a correlation between depression and excessive accumulation of iron ions in the brain. Consequently, exploring the regulation of ferroptosis as a potential therapeutic approach for depression holds significant promise for future drug research and development (DUAN et al., 2022).

In this comprehensive review, we searched for recent studies on depression and ferroptosis. PubMed, EMBASE, and MEDLINE scientific databases were searched individually and/or in combination using the following keywords: ferroptosis, depression, iron, antidepressants, mechanism. The original scientific papers, clinical trials, meta-analyses, and reviews written in English and published up to November 2023 addressing the above topics were enrolled. Case reports and letters were excluded, and 167 articles were ultimately included in the manuscript for review by reviewing the abstract, and full text. We analyzed the process of ferroptosis, explored the underlying pathological mechanisms of depression, and investigated potential connections between the ferroptosis signaling pathway and depression. Additionally, we reviewed relevant research on the modulation of ferroptosis signaling pathways as a potential approach for developing antidepressant interventions. Our aim was to establish a scientific foundation for future basic research and clinical applications in this field.

## 2 Overview of ferroptosis

**FIGURE 1:**
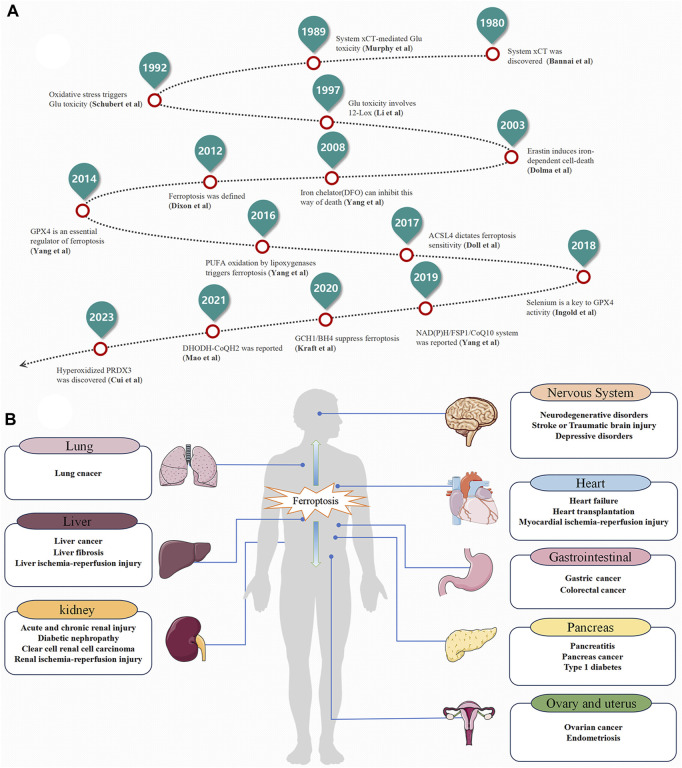
The Development History and Distribution of ferroptosis. **(A)** The Development History. **(B)** The Distribution of ferroptosis in the Human Body.

Ferroptosis is a form of cell death triggered by the excessive accumulation of iron-dependent lipid peroxides (LI et al., 2020). As early as 2003, Sonam Dolma discovered that erastin induced a novel form of cell death (DOLMA et al., 2003). In 2008, Wan Seok Yang and Brent R. Stockwell found that this type of cell death can be inhibited by iron-chelating agents (DFOM) and vitamin E (YANG and STOCKWELL, 2008). Later, in 2012, Brent R. Stockwell officially named this new form of cell death "ferroptosis” (Figure 1A). Notably, ferroptosis exhibits distinct characteristics in terms of cell morphology and biochemical indicators compared to other forms of regulated cell death (RCD). Unlike apoptosis, ferroptosis does not involve apoptotic bodies, cell shrinkage, or chromatin aggregation. Instead, it is characterized by reduced or vanished mitochondrial ridges, decreased cellular volume, and outer membrane rupture (DIXON et al., 2012; XIE et al., 2016). These unique features distinguish ferroptosis from known forms of cell death (Table 1). Over the past decade, significant progress has been made in the study of ferroptosis, establishing it as a field with great potential for development. Since ferroptosis can lead to damage and degenerative changes in various target organs, regulating ferroptosis signaling pathways holds great significance for improving related diseases (Figure 1B).

**TABLE 1:** The main morphological, biochemical, inducing factors and immune features of ferroptosis, apoptosis, autophagy and necroptosis.

	Ferroptosis	Apoptosis	Autophagy	Necroptosis
Morphological features	Significant ultrastructural changes mainly: mitochondrial crumpling, increased density of the bilayer membrane, rupture of the outer membrane, reduction or disappearance of the mitochondrial ridge (LI et al., 2020)	Cell membranes are vacuolated, nuclei are ruptured and crumpled, chromosomes condense, cell size decreases, and apoptotic vesicles form (LI et al., 2020)	Autophagic lysosome formation, cytoplasmic vesicularization (LI et al., 2020)	Cell membrane rupture, cytoplasmic and organelle swelling, cytoplasmic content efflux, chromatin condensation (LI et al., 2020)
Biochemical features	Iron accumulation, lipid peroxidation, decreased cystine uptake, decreased glutathione, increased NAPDH oxidation, release of arachidonic acid mediators, loss of mitochondrial membrane potential (XIE et al., 2016; LI et al., 2020)	Caspases activated, DNA fragmented, mitochondrial membrane potential reduced or absent, PS exposed (XIE et al., 2016; LI et al., 2020)	LC3-I is converted to LC3-II and autophagy substrates (e.g., p62) are catabolized (XIE et al., 2016; LI et al., 2020)	Decreased ATP levels, activation of RIP1, RIP3, and MLKL, release of DAMPs, and PARP1 hyperactivation (XIE et al., 2016; LI et al., 2020)
Inducing factors	Fe2+ accumulation, fatty acid enzyme catalysis	Normal gene regulation under physiological conditions	Lack of nutrition, microbial infections, organelle damageetc.	Severe pathologic injury
Immune features	Release of DAMPs pro-inflammatory (XIE et al., 2016)	Usually anti-inflammatory (XIE et al., 2016)	Usually anti-inflammatory (XIE et al., 2016)	Usually releases DAMPs pro-inflammatory (XIE et al., 2016)

NADPH: Nicotinamide adenine dinucleotide phosphate; Caspases: cysteinyl aspartate specific proteinase; PS: phosphatidylserine; DAMPs: damage associated molecular patterns; LC3-I: microtubule-associated proteins light chain 3-I; LC3-II: microtubule-associated proteins light chain 3-II; p62: prostacyclin-62; ATP: Adenosine triphosphate; RIP1: receptor-interacting protein 1; RIP3: receptor-interacting protein 3; MLKL: mixed-lineage kinase domain-like; PARP1: poly ADP-ribose polymerase-1.

## 3 Mechanism of ferroptosis

Ferroptosis primarily arises from the excessive accumulation of lipid reactive oxygen species (ROS) on the cell membrane due to intracellular metabolic dysregulation. Lipid peroxides and their secondary products, such as malondialdehyde (MDA) and 4-hydroxynonenal (4-HNE), can impair membrane integrity, proteins, and DNA, leading to increased membrane permeability and ultimately triggering ferroptosis (CAPELLETTI et al., 2020). The excessive accumulation of lipid peroxides can occur through two main mechanisms: enzymatic and non-enzymatic pathways (BATTAGLIA et al., 2020). The enzymatic mechanism involves the conversion of polyunsaturated fatty acids (PUFAs) into active lipid peroxides catalyzed by fatty acid enzymes. The other mechanism is attributed to iron metabolism disorders leading to the Fenton reaction. In recent years, additional pathways and targets related to ferroptosis have been identified, expanding the complexity of the regulatory mechanisms involving various signaling molecules and metabolic pathways.

The lipid metabolism pathway catalyzed by lipoxygenases (LOXs) is the primary enzymatic mechanism involved in promoting ferroptosis. Various sources of PUFAs undergo conversion into PUFA-CoA through acyl CoA synthase 4 (ACSL4). Subsequently, under the catalysis of lysophosphatidyltransferase 3 (LPCAT3), they are transformed into PUFA - phosphatidylethanolamine (PE) (PUFA-PE). These molecules are then oxidized by LOXs in the presence of unstable Fe^2+^, leading to the formation of phospholipid hydroperoxides (PLOOH) and promoting ferroptosis (FENG and STOCKWELL, 2018). In non-enzymatic pathways, the Fenton reaction mediated by Fe^2+^ plays a crucial role. Unstable chelated Fe^2+^ reacts with hydrogen peroxide (H2O2), generating highly reactive hydroxyl radicals (OH•), which then react with PUFAs, resulting in the formation of PLOOH and triggering ferroptosis (FENG and STOCKWELL, 2018).

### 3.1 Enzymatic mechanisms

#### 3.1.1 Lipid metabolism pathway

Among the enzymatic pathways involved in ferroptosis, LOXs play a paramount role. The three families of lipid oxidase (cyclooxygenases [COX], cytochromes P450 [CYPs], lipoxygenases [LOXs]) can convert free PUFA into lipid oxides, but it is the LOXs family that has the most significant impact on ferroptosis (FENG and STOCKWELL, 2018). LOXs are enzymes containing non-heme iron and exhibit specificity for the oxidation of PUFAs (XIE et al., 2022). Studies have demonstrated that PEs derived from arachidonic acid (AA) and adrenal acid (AdA) serve as crucial substrates for lipid peroxidation in ferroptosis (DOLL et al., 2017; KAGAN et al., 2017).

The process begins with PEs linked to free AA and AdA, which are converted into AA CoA and AdA-CoA through acyl CoA synthase long-chain family 4 (ACSL4). Subsequently, under the catalytic action of lysophosphatidyl acyltransferase member 3 (LPCAT3), AA-PE and AdA-PE are formed. Finally, these compounds are further converted into PLOOH through the combined catalysis of unstable Fe and LOXs, consequently triggering lipid peroxidation and cell ferroptosis (CHEN et al., 2022). Notably, ACSL4 and LPCAT3 play pivotal roles as driving factors for ferroptosis. ACSL4 is considered a sensitive marker for ferroptosis, as cellular ferroptosis can be induced by glutathione peroxidase 4 (GPX4)−/−, while cells with simultaneous knockout of both GPX4 and ACSL4 can survive normally (CAPELLETTI et al., 2020). Nevertheless, there is still ongoing debate regarding the key LOX subtypes driving ferroptosis in this process (FORCINA and DIXON, 2019). Studies have revealed that the LOX-15 complex, in conjunction with PE binding protein 1 (FEBP1), acts as a specific catalyst for AA/AdA-PEs, leading to lipid peroxidation and promoting cell ferroptosis (STOYANOVSKY et al., 2019). Additionally, another study identified edaravone (3-methyl-1-phenyl-2-pyrazolin-5-one, EDA) as an inhibitor of neuronal cell ferroptosis through downregulation of LOX-5 in neurons, thereby facilitating recovery after spinal cord injury (PANG et al., 2022).

Furthermore, investigations have demonstrated that the combination of nicotinamide adenine dinucleotide phosphate (NADPH)-cytochrome P450 reductase (POR) and nicotinamide adenine dinucleotide (NADH)-cytochrome b5 reductase (CYB5R1) allows electrons from NAD(P)H to react with O2, generating H2O2. Ultimately, this leads to the Fenton reaction with free iron ions, indirectly inducing lipid peroxidation and facilitating cell ferroptosis (YAN et al., 2021).

#### 3.1.2 Glutathione (GSH)-GPX4 pathway

The GSH-GPX4 pathway plays a crucial role in inhibiting lipid peroxidation, and the traditional approach to inducing ferroptosis involves inhibiting the synthesis pathway of GSH. In this pathway, GSH serves as a key antioxidant, and its synthesis primarily relies on intracellular cysteine (Cys) uptake through System Xc-. Cys is then reduced to serve as the direct source for GSH. While the System Xc-is considered the main source of Cys (LOU et al., 2022), studies suggest that Cys can also be synthesized through the sulfur transfer pathway when the System Xc-is inhibited (SHIMADA and STOCKWELL, 2015). The System Xc- and sulfur transfer pathway work cooperatively in Cys synthesis (ZHENG and CONRAD, 2020). However, dysregulation of the System Xc-still leads to Cys deficiency and GSH depletion in cells (LI FJ. et al., 2022).

GPX4, the fourth member of the GPX family containing selenium, plays a crucial role in inhibiting lipid peroxidation. GPX4 contains eight nucleophilic amino acids, including selenocysteine (Sec) (SHA et al., 2021), which is essential for its function (LEI et al., 2019). The unique amino acid sequence and structure of GPX4 establish it as a key regulatory factor in inhibiting ferroptosis (TURCHI et al., 2020; WU et al., 2022). Specifically, GPX4 utilizes reduced GSH as a critical substrate to convert GSH into oxidative GSH (GSSG) and PLOOH into fatty alcohols (PLOH), thus inhibiting the process of lipid peroxidation. In the catalytic cycle of GSH-GPX4, the active group -SeH of GPX4 is oxidized to -SeOH by PLOOH. GSH can then reactivate GPX4 by reducing -SeOH, releasing GSSG while maintaining the activity of GPX4 (LI FJ. et al., 2022). Consequently, inhibiting GSH synthesis has been shown in numerous studies to impair the activity of GPX4 (CHEN et al., 2019; YANG et al., 2020; QIN et al., 2021; YANG et al., 2021).

#### 3.1.3 Ferroptosis suppressor protein 1 (FSP1)-Coenzyme Q10 (CoQ10)-NADPH pathway

Alongside the GPX4 pathway, other pathways contribute to ferroptosis inhibition, notably the FSP1-CoQ10-NAD(P)H pathway and the methoxyphenate pathway. Recent studies propose that the FSP1-mediated ferroptosis defense mechanism can function as an independent parallel system (BERSUKER et al., 2019). This mechanism effectively suppresses ferroptosis even in the absence of GPX4 due to genetic deletion. In the FSP1-CoQ10-NADPH pathway, ferroptosis inhibitory protein 1 (FSP1), previously known as flavoprotein apoptosis-inducing factor mitochondrial 2 (AIFM2), acts as a potent ferroptosis inhibitor by reducing coenzyme Q (CoQ10) on the cytoplasmic membrane to panthenol (CoQH2) (SANTORO, 2020). CoQ10 serves as a lipophilic free radical scavenger and antioxidant, protecting the plasma membrane from oxidative damage caused by free radicals. Additionally, CoQ10 acts as a mobile lipid-soluble electron carrier, preserving the normal energy conversion of mitochondrial respiratory chains (SANTORO, 2020).

Nicotinamide adenine dinucleotide phosphate (NADPH), functioning as a dehydrogenase cofactor, is an essential reducing agent for clearing lipid hydroperoxides (STOCKWELL et al., 2017). FSP1 reduces CoQ10 to CoQH2 through NAD(P)H, which directly inhibits lipid peroxidation or indirectly promotes the regeneration of tocopherol free radicals (vitamin E), a natural chain-breaking antioxidant. This process eliminates lipid free radicals, thereby inhibiting lipid peroxidation (KAGAN et al., 2017). CoQ10 plays a vital role in this pathway, and when CoQ10 synthesis is inhibited, it results in increased lipid peroxidation (SANTORO, 2020). Although the precise source of CoQ10 synthesis remains unclear, most CoQ10 is synthesized within the mitochondria. Recent research identified a mitochondrial defense mechanism against ferroptosis mediated by dihydroorotate dehydrogenase (DHODH). In the absence of GPX4 activity, DHODH upregulates CoQH2 production to inhibit ferroptosis (MAO et al., 2021). Furthermore, studies have revealed that tetrahydrobiopterin (BH4), facilitated by GTP cyclohydrolase 1 (GCH1), effectively inhibits ferroptosis as a free radical scavenger. BH4 can suppress lipid peroxidation by generating CoQH2. Thus, it is plausible that two independent sources of CoQ10 production exist within cells (KRAFT et al., 2020; SANTORO, 2020; SOULA et al., 2020).

#### 3.1.4 Mevalonate (MVA) pathway

The MVA pathway is another important pathway involved in ferroptosis inhibition. It has significant roles in both the FSP1-CoQ10-NAD(P)H pathway and the GSH-GPX4 pathway. The MVA pathway starts with acetyl CoA, which undergoes a series of reductase reactions, including 3-hydroxy-3-methylglutaryl CoA reductase (HMGCR), leading to the production of MVA. Under the action of coenzymes like methylglutarate kinase (MVK), MVA further produces isoamyl pyrophosphate (IPP) (CHEN et al., 2020). IPP is an intermediate molecule in various biomolecules involved in the regulation of ferroptosis (ZHENG and CONRAD, 2020). For example, IPP can generate farnesyl pyrophosphate (FPP) through the activity of phosphofarnesyl synthase. Subsequently, FPP can be converted to squalene by squalene synthase (SQS). Squalene then undergoes cyclization by squalene cyclase to produce cholesterol. However, when SQS is inhibited, FPP bypasses the cholesterol synthesis pathway and instead converts to CoQ10 (CHEN et al., 2020), thereby inhibiting ferroptosis. Studies have demonstrated that FIN56, a known ferroptosis inducer, can activate SQS, leading to reduced CoQ10 synthesis (SHIMADA et al., 2016).

The MVA pathway also plays a crucial role in GPX4 synthesis. In the selenium-containing GPX4 synthesis pathway, the insertion of Sec is essential for GPX4 to exert its antioxidant activity. This process is facilitated by the catalytic integration effect of IPP (WU et al., 2022). FIN56, as a ferroptosis inducer, can inhibit the integration of Sec on the GPX4 catalytic subunit, thereby reducing the expression of GPX4’s antioxidant activity (OU et al., 2022).

#### 3.1.5 Glutamine (Gln) metabolism pathway

The Gln metabolism pathway is another important pathway involved in the regulation of ferroptosis. Gln, a key amino acid in the human body, plays a crucial role in various biological processes and energy production within mitochondria, such as the tricarboxylic acid (TCA) cycle (KOPPULA et al., 2021). Gln is primarily taken up by cells through SLC1A5 and is then broken down by glutaminase (GLS) into glutamic acid. The converted glutamic acid enters the TCA cycle as α-ketoglutaric acid (α-KG) with the help of enzymes like glutamate (GLU) dehydrogenase (GLUD1) (LI S. et al., 2023), regulating lipid synthesis and the ferroptosis process (YAO et al., 2021). GLS, a key enzyme in Gln breakdown, consists of two subtypes: GLS1 and GLS2. Studies have demonstrated that GLS2 is the essential subtype for mitochondrial regulation of ferroptosis (GAO et al., 2015; SUZUKI et al., 2022). GLS2 facilitates the production of glutamic acid, which enters mitochondria and is further converted to α-KG by aspartate aminotransferase (GOT) and GLUD1. This process maintains energy metabolism and component transformation in the TCA cycle (YAO et al., 2021). The glutamic acid derived from Gln breakdown in the TCA cycle induces structural and functional changes in mitochondria, including depolarization of the mitochondrial membrane potential and reduced activity of the mitochondrial electron transfer chain. These changes contribute to the accumulation of lipid ROS, ultimately leading to ferroptosis (SONG et al., 2023). Therefore, modulation of the Gln metabolism pathway can regulate the sensitivity of cells to ferroptosis (KANG et al., 2019; SUZUKI et al., 2022).

Downregulation of solute carrier family 7 member 11 (SLC7A11), a component of System Xc-, has been shown to induce ferroptosis (CHEN et al., 2021). Recent studies have revealed that increased expression of SLC7A11 in tumor cells leads to excessive cystine uptake for GSH synthesis and loss of GLU (YAO et al., 2021), resulting in an imbalance in the Gln metabolism pathway. By inhibiting SLC7A11 or depriving cells of Cys, the glutamic acid produced from Gln breakdown can bypass System Xc- and enter the TCA cycle, leading to the accumulation of lipid ROS and ultimately triggers ferroptosis. Targeting this pathway provides new potential strategies for cancer treatment based on ferroptosis (KOPPULA et al., 2021).

### 3.2 Non-enzymatic mechanisms

#### 3.2.1 Disorders of iron ion metabolism

As mentioned previously, ferroptosis is primarily triggered by iron-dependent lipid peroxidation. Iron ions are crucial for human physiological processes. Under normal conditions, systemic iron homeostasis relies on the coordinated expression of transferrin (TF), ferroportin 1 (FPN1), transferrin receptor 1 (TfR1), and liver-produced iron regulatory proteins (ROCHETTE et al., 2022). Two forms of iron ions exist in the human body: Fe^2+^ and Fe^3+^. When there is an imbalance in iron homeostasis, these ions participate in oxidation-reduction reactions, resulting in the production of peroxy free radicals and ultimately leading to ferroptosis (LOU et al., 2022).

During this process, extracellular Fe^3+^ binds to TF and is recognized by TfR1 before being internalized into the cell. Subsequently, within vesicles, prostate transmembrane epithelial 3 antigen antibody (STEAP3) facilitates the reduction of Fe^3+^ to Fe^2+^. The transported Fe^2+^ from the vesicles enters the cytoplasm either through divalent metal transporter 1 (DMT1) and zinc iron regulatory protein family 8/14 (ZIP8/14), or it binds to ferritin, storing it in unstable iron pools (LIPs). Free Fe^2+^ in the cytoplasm is susceptible to the Fenton reaction, leading to lipid peroxidation. Additionally, ferritin bound to Fe^2+^ in LIP undergoes autophagic degradation, releasing free Fe^2+^ mediated by nuclear receptor coactivator 4 (NCOA4) upon entering the lysosome (CHEN et al., 2022). This initiates the Fenton reaction and promotes lipid peroxidation. FPN serves as the sole protein responsible for transporting Fe^2+^ out of cells, thereby reducing intracellular Fenton reactions and inhibiting ferroptosis (SUN et al., 2022). Furthermore, studies have shown that Fe^2+^ exported via FPN can be converted to less oxidizing Fe^3+^ through the action of ceruloplasmin (CP). Depletion of CP leads to the accumulation of intracellular Fe^2+^, thus inducing ferroptosis (SHANG et al., 2020).

Moreover, unstable iron within LIP can bind to ferritin (including both ferritin light chain and ferritin heavy chain) and transform into Fe^3+^ for storage in lysosomes, effectively inhibiting lipid peroxidation. In instances of iron deficiency, iron bound to ferritin heavy chain (FTH) can be released into LIP with the aid of NCOA4. Free Fe^2+^ within LIP is crucial for mitochondrial function (FUJIMAKI et al., 2019). The influx of Fe^2+^ from unstable iron pools into mitochondria not only triggers the Fenton reaction leading to ferroptosis, but also serves as a substrate for Fe-S cluster synthesis. The stability of Fe-S clusters plays an indispensable role in maintaining the normal functioning of electron transfer chains (ETC.) and the TCA cycle (FANG et al., 2023). Damage to, ETC can also result in lipid peroxidation and subsequent ferroptosis within cells (JAVADOV, 2022; DONG et al., 2023).

#### 3.2.2 System Xc-pathway

**FIGURE 2:**
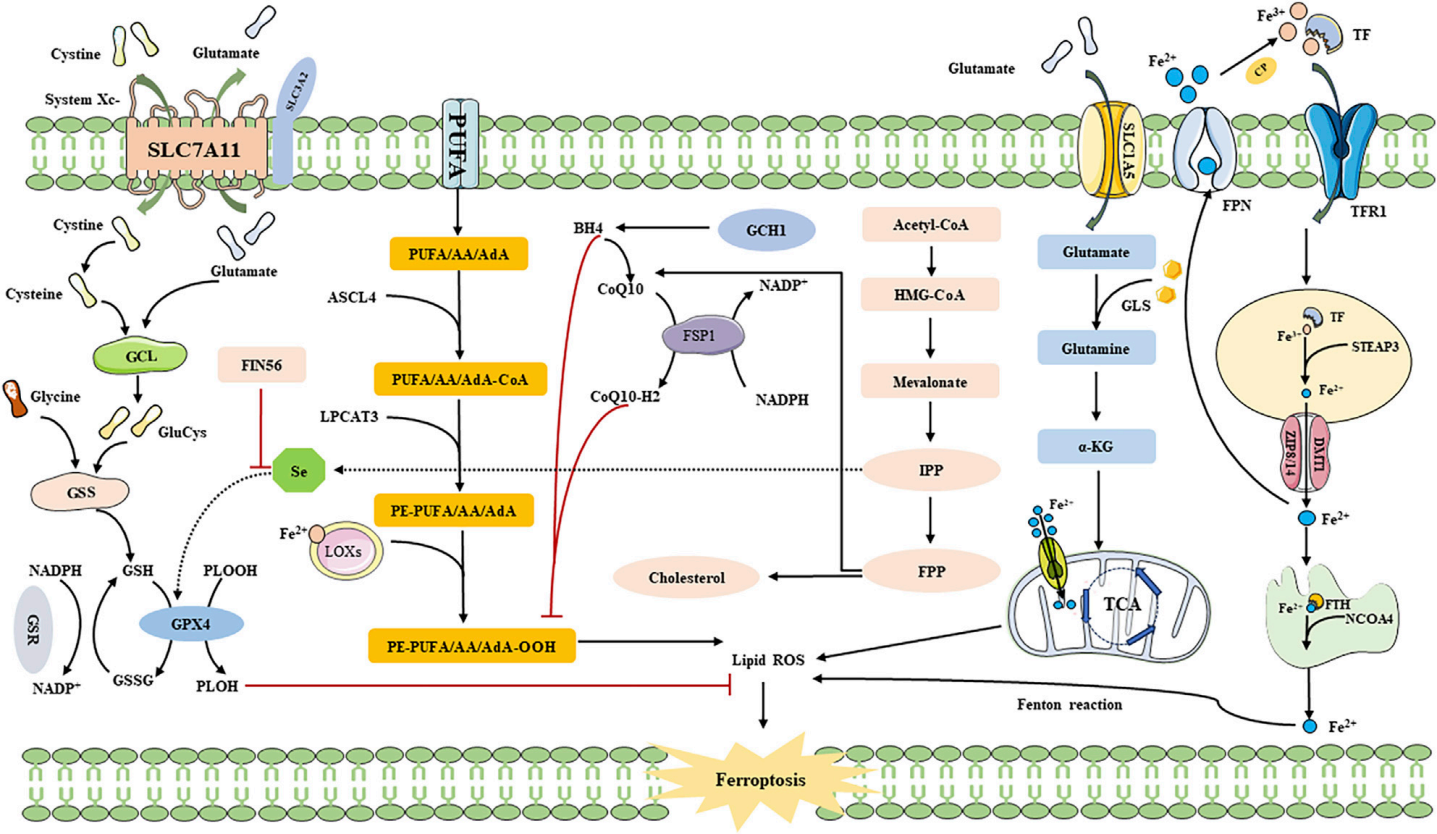
The Mechanism of ferroptosis. SLC3A2: solute carrier family 3 member 2; SLC1A5: solute carrier family 1 member 5; SLC7A11: recombinant solute carrier family 7 member 11; GCL: Glutamate-cysteine ligase; GSS: Glutathione synthetase; GSSG: oxidative Glutathione; PLOOH: phospholipid hydroperoxides; PLOH: fatty alcohols; AA: arachidonic acid; AdA: adrenal acid; LOXs: lipoxygenases; CoQ10: Coenzyme Q10; DMT1: Divalent metal transporter 1; Fe^2+^: Ferrous iron; Fe^3+^: Ferric iron; FPN: Ferroportin; FSP1: Ferroptosis suppressor protein 1; FTH: Ferritin heavy chain; GSR: Glutathione reductase; GPX4: Glutathione peroxidase 4; GSH: Glutathione; LPCAT3: Lysophosphatidylcholine acyltransferase 3; NCOA4: Nuclear receptor coactivator 4; PUFA: polyunsaturated fatty acid; ROS: Reactive oxygen species; TF: Transferrin; GLS: glutaminase; LPCAT3: lysophosphatidylcholine acyltransferase 3; TfR1: transferrin receptor 1; TCA: citric acid cycle; α-KG: α-ketoglutarate; NADPH: nicotinamide adenine dinucleotide phosphate; CoQ10H2: ubiquinol; GCH1: guanosine triphosphate cyclohydrolase 1; STEAP3: six-transmembrane epithelial antigen of prostate 3; ZIP8/14: zinc iron regulatory protein family 8/14; ACSL4: acyl-CoA synthetase long-chain family member 4; FIN56: ferroptosis inducer; Se: Selenium; HMG-CoA: 3-Hydroxy-3-Methyl-Glutaryl-CoA; IPP: isoamyl pyrophosphate; FPP: farnesyl pyrophosphate.

The System Xc-pathway is facilitated by a chloride ion-dependent Glu/Cys transporter protein located on the plasma membrane. This protein consists of solute carrier family 7 member 11 (SLC7A11) and solute carrier family 3 member 2 (SLC3A2), which are interconnected by disulfide bonds (LI FJ. et al., 2022). System Xc-operates based on a concentration gradient of Glu/Cys both inside and outside the cell, allowing for Cys intake into the cell in a 1:1 ratio while Glu is excreted (ISHII et al., 1987; CONRAD and SATO, 2012; LI et al., 2020; WU X. et al., 2021). Several biomolecular synthesis processes occur following the uptake of Cys via System Xc-, leading to the synthesis of GSH. In this process, ingested Cys is catalyzed by thioredoxin reductase 1 (TrxR1) to convert it to Cys, which is then combined with glutamic acid (Glu) through the catalytic action of GLU Cys ligase (GCL) to form γ-glutacyl-L-Cys. Ultimately, this compound is directly utilized in the biosynthesis of GSH along with glycine, thereby inhibiting ferroptosis (LIN et al., 2020; LI FJ. et al., 2022; LIU et al., 2022). Among these steps, the transport of Cys through the amino acid transport system is primarily regulated by SLC7A11 (LI FJ. et al., 2022). Thus, inhibiting the expression of SLC7A11 can increase cellular sensitivity to ferroptosis (JENNIS et al., 2016; NISHIZAWA et al., 2020; WANG et al., 2020). Intracellular Cys can also be acquired through the sulfur transfer pathway and neutral amino acid transporters (CONRAD and SATO, 2012; HAYANO et al., 2016). This indicates that the induction of ferroptosis by targeting the Cys source is not limited to a singular pathway. However, when ferroptosis inducers like Erastin and its analogues inhibit System Xc- (SATO et al., 2018), intracellular Cys depletion still occurs, leading to reduced GSH synthesis and eventual lipid peroxidation. Therefore, targeting the System Xc-pathway is crucial in modulating GSH synthesis (SHAO et al., 2022) (Figure 2).

## 4 Depression and ferroptosis

### 4.1 Iron deposition in depression

Researchers have discovered iron deposition in various individuals with depression, and the dysregulation of iron homeostasis in the brain has been strongly linked to depression (ZHU et al., 2016; WANG et al., 2019; KNYSZYŃSKA et al., 2020; BERTHOU et al., 2021). Initially, different extracellular Fe^3+^ ions are recognized by TfR1 and enter the cytoplasm, where they are then converted into Fe^2+^ under the catalytic influence of STEAP3. Excessive ferrous ions can easily trigger the Fenton reaction, leading to ferroptosis. Therefore, upregulating TfR1 expression increases cellular susceptibility to ferroptosis (YU et al., 2021). Towards the end of the 20th century, a close association between increased TfR1 expression and depression was identified in patients with this condition (MAES et al., 1995a). This suggests that individuals with depression may exhibit a higher propensity for the excessive accumulation of iron ions, resulting in neuronal ferroptosis (MAES et al., 1995b; LIANG et al., 2020). However, some researchers further demonstrated, through a cross-sectional study, that ferroptosis is not related to the severity of depression (ZHANG et al., 2019). Thus, iron deposition in the gray matter of the brain may serve as a potential biomarker for depression (YAO et al., 2017). Moreover, the relationship between depression and iron deposition has also been established through animal experiments. Chang et al., using proteomic techniques, compared the expression differences of TF and TfR1 in the brains and peripheral blood of normal mice and chronic social defeat stress (CSDS)-induced depression mouse models. The results indicated higher expression levels of TF and TfR1 in the peripheral blood and brain of CSDS-induced depression mouse models compared to the control group (CHANG et al., 2022). Further studies have demonstrated a strong correlation between the onset of depression and the reduction of hippocampal neuronal synapses, which is attributed to the upregulation of TfR1 and downregulation of FTL caused by nuclear factor erythroid-2 related factor 2 (Nrf2) deficiency (ZENG et al., 2023). These findings align with the research conducted by Wang et al., whose team elucidated the association between iron deposition in the hippocampus and depression, along with confirming the reduction of FTH induced by lipopolysaccharide (LPS) in depressed mice (WANG et al., 2021a).

### 4.2 Potential pathologic link between depression and ferroptosis

#### 4.2.1 Iron deposition and neuroinflammation

Neuroinflammation refers to the immune response of the central nervous system (CNS) mediated mainly by microglia and astrocytes in the hippocampus (LEE and HYUN, 2023). Microglia, known for their high iron content (UZUNGIL et al., 2022), have been implicated in depression associated with abnormal glial activation and iron overload, suggesting a potential connection between iron and neuroinflammation (ZENG et al., 2023). However, the precise mechanism by which iron overload disrupts neurotransmitter homeostasis and induces anxiety and depressive behaviors remains unclear (UZUNGIL et al., 2022).

Research has shown that signal transduction involving brain-derived neurotrophic factor (BDNF) plays a crucial role in synaptic plasticity in depression, and the downregulation of BDNF may contribute to neurotoxic effects (SHKUNDIN and HALARIS, 2023). In this context, Li et al. demonstrated that iron overload may downregulate BDNF via the iron urin BDNF pathway, leading to hippocampal nerve damage (LI J. et al., 2023). Furthermore, Gao et al. showed in a chronic unpredictable mild stress (CUMS) mouse model that iron deposition in microglia within the hippocampus is closely associated with neuronal degeneration and death (GAO W. et al., 2019). Additionally, Zeng et al. highlighted the significant role of Nrf2 as an anti-inflammatory factor in regulating iron deposition and neuroinflammatory responses in depression (ZENG et al., 2023). In a comparative study using hippocampal proteomics, Cao et al. observed distinct protein expression differences between normal mice and CUMS model mice, indicating pronounced activation of neuronal necrosis and iron deposition in the hippocampus, thus promoting the occurrence of depression (CAO et al., 2021). Recently, Zhang et al. discovered that expression levels of various inflammatory factors significantly increased in CUMS model mice, while treatment with the iron chelating agent deferoxamine (DFO) effectively reversed this neuropathological change (ZHANG W. et al., 2022). Collectively, these findings suggest a potential link between the neurotoxicity induced by iron overload and the development of depression.

#### 4.2.2 Iron deposition and mitochondrial dysfunction

Since the discovery of ferroptosis in 2012, mitochondria have been proposed to play a crucial role in regulating erastin-induced ferroptosis (FANG et al., 2023). Multiple membrane iron transporters (MFRNs) located on the mitochondrial membrane facilitate the distribution of iron within mitochondria and the cytoplasm. When mitochondrial iron overload occurs, it leads to the generation of a substantial amount of mtROS, resulting in cellular ferroptosis (FUHRMANN and BRÜNE, 2022). Two potential mechanisms for mitochondrial ferroptosis have been suggested (FANG et al., 2023). Firstly, an imbalance in mitochondrial iron homeostasis and an elevation in Fe^2+^ levels promote ROS production via the Fenton reaction, ultimately inducing cell ferroptosis. Secondly, as iron serves as a key regulator of oxidative phosphorylation (OXPHOS), disruption of iron metabolism can lead to impaired electron transfer, lipid peroxidation, and subsequent induction of ferroptosis. Mitochondria contain a significant amount of iron, primarily involved in heme synthesis, Fe-S cluster biosynthesis, and storage within mitochondrial-specific ferritin (FtMt), which maintains the structure and function of the electron transport chain (ETC.) and TCA cycle. Disturbances in iron metabolism can significantly cause structural changes and dysfunction of mitochondria (CHENG et al., 2022; DONG et al., 2023). ROS produced by mitochondrial dysfunctionis essential for ferroptosis induction (GAO M. et al., 2019). Numerous studies have demonstrated a close association between depression and oxidative stress, as well as disruptions in the, ETC within mitochondria (SONG et al., 2023; XU et al., 2023). In recent years, research has increasingly focused on the mechanism of ferroptosis in patients with depression. Adenosine triphosphate (ATP), predominantly produced by mitochondria, has been found to exhibit a positive correlation with the occurrence of depression (INCZEDY-FARKAS et al., 2014; CHANG et al., 2015; BRYMER et al., 2018; WANG et al., 2018). Kondo et al. further confirmed this relationship using magnetic resonance spectroscopy (MRS) (KONDO et al., 2011). When mitochondrial iron metabolism disorders occur, excessive free iron ions can impair the, ETC and subsequently inhibit ATP production (BATTAGLIA et al., 2020). Romano et al. reviewed the potential role of 4-HNE, a product of ferroptosis (CAPELLETTI et al., 2020), in the pathogenesis of mental disorders such as bipolar disorder and depression, along with lipid peroxidation (ROMANO et al., 2017). Mitochondria, being the primary source of cellular ROS, may also be involved in modulating depression via the ferroptosis signaling pathway (BANSAL and KUHAD, 2016). Based on the available evidence, disturbances in mitochondrial iron metabolism can contribute to the development of depression by influencing mitochondrial function and structure (ZUO et al., 2022; SONG et al., 2023).

#### 4.2.3 Iron deposition and gut flora

The brain-gut axis (GBA) refers to a bidirectional signaling network involving the brain, gut, and gut microbiota. This network operates through neuroendocrine, immune regulation, and microbial molecules, connecting emotional and cognitive activities in the brain with physiological and pathological changes in the peripheral gut (KANIA et al., 2023). The occurrence of depression and bipolar disorder may be associated with intestinal microbiota and dysfunction in the bidirectional communication within the CNS (GÓRALCZYK-BIŃKOWSKA et al., 2022). Remarkably, imbalances in serum iron homeostasis can induce both intestinal microbiota imbalance and inflammation, leading to stress responses in the brain. Consequently, the expression of IL-6 is promoted, which triggers glial cells to release hemodulin, isolating excess iron in neurons. However, this process can unexpectedly induce oxidative stress response, resulting in neuronal damage (HOUSER and TANSEY, 2017; KANIA et al., 2023). Furthermore, studies have elucidated the potential connection between the IL-6-mediated signaling pathway and the pathogenesis of depression (TING et al., 2020). Additionally, as previously mentioned, iron overload may contribute to depression by downregulating the expression of BDNF (LI J. et al., 2023; SHKUNDIN and HALARIS, 2023). Recent research has demonstrated that alterations in gut microbiota distribution, according to the brain-gut axis mechanism, can modulate BDNF expression, thereby influencing the onset of depression (LIAQAT et al., 2022). Moreover, a meta-analysis revealed decreased levels of *Corprococcus* and *Faecalibacterium* bacteria in the intestines of individuals with depression compared to healthy individuals. Notably, intervention with probiotics resulted in improved depression symptoms. Taken together, these findings suggest that ferroptosis may inhibit the damage caused by neuroinflammation through cross talk among the brain-gut axis mechanism, gut microbiota, and CNS (WANG et al., 2023a; KANIA et al., 2023). Thus, it holds promise as a potential target for the diagnosis and treatment of depression.

#### 4.2.4 Iron deposition and autophagy

Autophagy is a natural and RCD process that facilitates the degradation of damaged organelles, pathogens, and other biological components (such as proteins, lipids, DNA, and RNA) within cells. It serves as a survival mechanism in response to stress (LIU et al., 2020). The relationship between autophagy and ferroptosis remains unclear. Recent studies have proposed that ROS triggered by the ferroptosis inducer erastin can induce autophagy in cells, and autophagy, in turn, can regulate ferroptosis by degrading cellular ferritin (PARK and CHUNG, 2019). Zuo et al. demonstrated that oxidative stress, mediated by ROS, plays a crucial role in depression induction, and Nrf2 activation can mitigate oxidative stress by simultaneously regulating multiple potential mechanisms, including autophagy and ferroptosis (ZUO et al., 2022). Furthermore, mitochondrial autophagy has been found to be significant in depression (TRIPATHI et al., 2021), and Nrf2 activation can directly modulate mitochondrial autophagy (MURATA et al., 2015). From the perspective of ferroptosis, the ferroptosis pathway may also hold promise as a potential mechanism for treating depression (ZUO et al., 2022). Autopsy results from patients with depression revealed decreased levels of GSH and GPX in the brain (GAWRYLUK et al., 2011). Wigner et al. identified a potential association between GPX4 gene polymorphism and depression regulation (WIGNER et al., 2018). Additionally, it has been demonstrated that most genes involved in ferroptosis are directly or indirectly regulated by Nrf2 (HARADA et al., 2011; DODSON et al., 2019). Dang et al. investigated the effects of EDA and showed its ability to reduce oxidative stress and ferroptosis through the Silent information regulatory factor 2 homologous protein 1 (Sirt1)/Nrf2/Heme oxygenase-1 (HO-1)/Gpx4 pathway, thereby inhibiting the development of depression (DANG et al., 2022). These findings provide evidence of crosstalk between autophagy and ferroptosis in the pathological mechanism of depression, which may serve as a potential therapeutic avenue for depression.

## 5 Treating depression by inhibiting ferroptosis

### 5.1 Traditional Chinese medicine

#### 5.1.1 Active ingredients of traditional Chinese medicine

Allicin, a prominent active compound in Chinese herbal garlic, has been found to possess neuroprotective effects (CHEN et al., 2014). Gao et al. demonstrated that Allicin can alleviate depressive behavior in the CSDS model mice by inhibiting NLRP3 inflammasomes. Increased concentration of DMT1 in hippocampal neurons during the chronic phase of inflammation indicates iron deposition, suggesting a potential link between Allicin and ferroptosis as one of the mechanisms for treating depression (GAO W. et al., 2019).

Saikosaponin B2 (SSB2), derived from the Chinese herbal medicine Radix Bupleuri, has a long history of being used to treat depression, as documented in the TCM classic “*Taiping Huimin HeJiJu Fang*” (YANG et al., 2017). The antidepressant and neuroprotective effects of Radix Bupleuri have been confirmed through modern research (TONG et al., 2024). Wang et al., using the CUMS mouse model and ferroptosis mouse model, demonstrated that SSB2 inhibits Toll-like receptor 4 (TLR4)/nuclear factor kappa-B (NF-κB) pathway-mediated ferroptosis to improve depression-induced microglia activation. This leads to inhibition of pro-inflammatory cytokines IL-1β, IL-6, and TNF-α. However, the therapeutic effect of SSB2 on depression is blocked when GPX4 is knocked out, indicating that SSB2 acts through a GPX4-dependent manner in mediating TLR4/NF-κB pathway, exerting its anti-ferroptosis and anti-neuroinflammatory roles (WANG et al., 2023b).

Lycium barbarum glycopeptide (LbGp), a carbohydrate conjugate composed of proteins and monosaccharides, is a major active component of Chinese herbal medicine *Lycium barbarum* (PENG et al., 2001). Recent studies have shown that goji berry polysaccharides effectively alleviate depression (FU et al., 2021; LI X. et al., 2022). Dai et al. observed in their study using chronic restraint stress (CRS) model mice that GPX4 knockout-induced ferroptosis disrupts cortical function, leading to increased depression and anxiety in mice. However, treatment with LbGp can attenuate the anxiety caused by ferroptosis by increasing superoxide dismutase (SOD) activity and inhibiting the elevation of GSH and MDA levels (DAI et al., 2023). Gallic acid (3,4,5-trihydroxybenzoic acid) is an active ingredient extracted from Chinese herbal medicine found in various plants, such as green tea and gallnuts (Al Zahrani et al., 2020). Recent studies have revealed that the phenolic hydroxyl group of gallic acid has the ability to clear ROS and disrupt the cycle of new free radicals, thereby exhibiting anti-inflammatory properties (TEIXEIRA et al., 2013). Yang et al. discovered that gallic acid can effectively treat depression development by inhibiting the P2X7 regulatory ferroptosis signaling pathway in chronic contractile injury (CCI) model rats and CUMS model rats. Inhibition of P2X7 expression leads to downregulation of TNF-α, NF-κB expression levels, and STAT3 phosphorylation. Consequently, the expression of GSH and GPX4 increases, which suppresses ROS accumulation and the generation of MDA, ultimately alleviating the pain and depressive symptoms in CCI and CUMS rats through the inhibition of ferroptosis (YANG R. et al., 2023). Silybin, another primary active ingredient derived from Chinese herbal medicine Silybin, has been shown to possess neuroprotective effects as well as antidepressant effects (SONG et al., 2018; LIU et al., 2021). Liu et al. discovered that silybin reduces neuroinflammation mediated by interferon gene stimulating factor (STING) by downregulating the ferroptosis signaling pathway. This leads to an improvement in depressive behavior in an Alzheimer’s disease mouse model. Hippocampal neurons (HT-22) treated with streptozotocin (STZ) exhibit dysregulation of the p53/SLC7A11/GSH/GPX4 pathway, resulting in excessive ROS accumulation and ferroptosis activation, which subsequently triggers the cellular immune response via the STING signaling pathway. This pathological mechanism induces the release of various inflammatory factors, leading to depressive behavior in mice. Silybin treatment can reverse this pathological change by inhibiting the ferroptosis signaling pathway (LIU et al., 2023) (Table 2).

**TABLE 2:** Regulation of ferroptosis by the active compounds of traditional Chinese medicine.

Active compounds of TCM	Chemical structure	Molecular formula	CAS number	Moulding method	Animal or cell type	Dosage of drugs used	Behavioral testing evaluation	Antidepressant mechanisms	References
Allicin	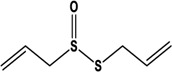	C_6_H_10_OS_2_	539-86-6	CSDS	C57BL/6J mice, CD-1 mice	2, 10, 50 mg/kg	SPT, FST, SIT	Amelioration of Neuroinflammation, Abnormal Iron Accumulation, Oxidative Stress, and Neuronal Apoptosis via Inhibition of the NLRP3 Pathway in the Hippocampus Ameliorates Depression-Like Behavior	GAO et al. (2019a)
Saikosaponin B2	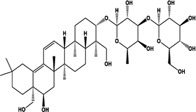	C_42_H_68_O_13_	58,316-41-9	CUMS	ICR mice of SPF grade	5, 10 mg/kg	SPT, OFT, FST	Inhibition of ferroptosis, neuroinflammation via TLR4/NF-κB pathway in a GPX4-dependent manner ameliorates depression	WANG et al. (2023b)
Gallic Acid	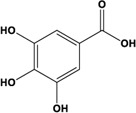	C_7_H_6_O_5_	149-91-7	CUMS、CCI	Sprague−Dawley rats	100 mg/kg	PBT, SPT, FST, OPT	Modulation of GSH/GPX4 signaling pathway in ferroptosis by inhibition of P2X7 ameliorates depression	YANG et al. (2023a)
Silibinin	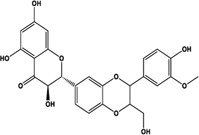	C_25_H_22_O_10_	802,918-57-6	STZ	Sprague-Dawley rats	25, 50, 100 mg/kg	NORT, EPMT, FST, SPT	Inhibition of STING signaling pathway activation through modulation of p53/SLC7A11/GSH/GPX4 signaling pathway to ameliorate depression	LIU et al. (2023)

CCI: chronic constrictive injury; STZ: streptozotocin; NLRP3: NOD-like receptor pyrin domain-containing 3; NF-Κb: Nuclear factor-kappa B; TLR4: Toll-like receptor 4; SLC7A11: Solute Carrier Family 7 Member 11; PBT: pain behavioral test; SIT: social interaction test; p53: tumor suppressor protein.

#### 5.1.2 TCM compounds

Currently, there is limited research on the modulation of the ferroptosis signaling pathway in TCM prescriptions for treating depression. Recent studies employing metabolomics and nuclear MRS have revealed the potential antidepressant effects of Xiaoyao powder (consisting of *Radix Bupleuri*, *Poria*, *Rhizoma Zingiberis Recens*, *Radix Angelicae Sinensis*, *Radix* Paeoniae Alba, *Rhizoma Atractylodis Macrocephalae*, *Radix Glycyrrhizae*, and *Herba Menthae Haplocalycis*) on CUMS rats (LIU et al., 2019). Jiao et al. found that Xiaoyao powder ameliorates depression-like behavior in CUMS model mice by modulating the ferroptosis signaling pathway mediated by the hippocampus. The antidepressant mechanism of Xiaoyao powder may involve the upregulated expressionsof PEBP1 and extracellular regulatory protein kinase 1/2 (ERK1/2), and modulation of GPX4 within the ferroptosis signaling pathway. These findings suggest that Xiaoyao powder may alleviate depressive behavior in mice through mediating PEBP1-GPX4 interactions (JIAO et al., 2021).

Furthermore, Dihuang Yinzi (composed of *Radix Rehmanniae* Praeparata, *Radix Ophiopogonis*, *Caulis Dendrobii*, *Fructus Corni*, *Fructus Schisandrae Chinensis*, *Herba Cistanches*, *Radix Morindae Officinalis*, *Radix Aconiti Lateralis Praeparata*, *Cortex Cinnamomi*, *Poria*, *Rhizoma Acori Tatarinowi*i, *Radix Dolygalae*, *Herba Menthae*, *Rhizoma Zingiberis Recens*, and *Fructus Jujubae*) has been found to possess neuroprotective effects (ZHANG et al., 2017). Moreover, Zhou et al. observed that Dihuang Yinzi can mitigate neural damage caused by post-stroke depression (PSD) in rats by regulating the ferroptosis signaling pathway. The active extract of Dihuang Yinzi modulates the P53/SLC7A11 pathway and upregulates the expression of SLC7A11 protein through promotion of P53 ubiquitination, consequently increasing GPX4 expression. This intervention effectively inhibits ferroptosis in PSD, improving depressive symptoms and cognitive impairment (YANG Z. et al., 2023).

### 5.2 Chemicals with antidepressant effects

In addition to extracts and active ingredients from TCM compounds, several other chemicals have shown potential in regulating ferroptosis pathways to ameliorate depression (WANG et al., 2021b; DANG et al., 2022; UZUNGIL et al., 2022; Wang et al., 2022; Zhang et al., 2022; Li et al., 2023). Deferiprone (DFP), a commonly used iron chelating agent, demonstrates the ability to cross the blood-brain barrier (BBB) and transfer iron from DFP-Fe to TF for the treatment of serum iron overload. It has been proven effective with low toxicity in improving depressive-like behavior by reducing brain iron levels and tau protein levels (Rao et al., 2020; SUN et al., 2023). Furthermore, Uzungil et al. propose that DFP may exert its antidepressant effect through the lateral amygdala and lateral septum, potentially associated with changes in the unstable iron pool in the brain (UZUNGIL et al., 2022).

Edaravone (EDA) is a highly biologically active free radical scavenger known for its antioxidant, anti-inflammatory, and neuroprotective effects (SRIRAM et al., 2016). It is commonly used in the treatment of acute cerebrovascular diseases. Studies indicate that EDA may improve corticosterone-induced depression-like behavior in mice by modulating the Fkbp5, Comt, Adora1, and Slc6a15 genes (HERBET et al., 2019). Dang et al. found that EDA can alleviate depression and anxiety-like behavior through the Sirt1/Nrf2/HO-1/Gpx4 pathway using the CSDS mouse model. SIRT1 affects downstream Nrf2. HO-1, a key stress-inducing protein targeted by Nrf2, participates in GPX4 synthesis. Both Nrf2 and HO-1 inhibit ferroptosis and the activation of pro-inflammatory cytokines (IL-1β, IL-6, and TNF-α), thereby improving depressive-like behavior (DANG et al., 2022).

Eicosapentaenoic acid (EPA) and docosahexaenoic acid (DHA) are essential components of Omega-3 PUFAs (n-3-PUFAs), known to improve depression by reducing inflammation and enhancing the expression of neurotrophic factors (PENG et al., 2020). Moreover, a potential association has been found between decreased peripheral blood concentration of n-3-PUFAs, particularly EPA and DHA, and the pathogenesis of depression (Song et al., 2016). Wang et al. conducted a study using the pentylenetetrazole (PTZ) provocation model and found that both DHA and EPA can alleviate depressive behavior in mice by inhibiting ferroptosis and neuroinflammation. Following PTZ treatment, there was a significant increase in the expression of iron regulatory protein (IRP) 1 (IRP1), IRP2, and TfR1 in mice, while FPN1 and FTH1 showed significant reductions. Additionally, Western blot analysis revealed a decrease in the protein expression of GPX4, System Xc-, Nrf2, and HO-1 in mice, but EPA and DHA were able to reverse these pathological changes, mitigating inflammation and iron deposition, and ultimately improving depressive behavior in mice (Wang et al., 2022).

Hydrogen sulfide (H_2_S), a gaseous signaling molecule, plays various roles such as neuronal transmission, smooth muscle relaxation, and cell protection against oxidative stress. It is produced by enzymes including β Synthases, Cystine γ Lyase, 3-mercaptopyruvate thiotransferase, and Cys aminotransferase (WU et al., 2018). Recent research has discovered that H2S can inhibit depression-like behavior induced by CRS through upregulation of adiponectin (Q et al., 2018). Furthermore, Wang et al. utilized a model of type 1 diabetes mice induced by STZ to demonstrate that H2S can alleviate depression-like behavior in these mice by inhibiting the inflammatory response and the ferroptosis signaling pathway. In the STZ model, ferroptosis occurs alongside GPX4 inactivation, oxidative stress, and System Xc-inhibition. NaHS, as the primary donor of H2S, can enhance GPX4 activity and increase the expression of SLC7A11 and Cys β-synthase (CBS), thereby reducing the levels of MDA and ROS, ultimately inhibiting ferroptosis. Additionally, NaHS can suppress the inflammatory response by upregulating the expression of sirtuin 6 (Sirt6), leading to reduced acetylation of histone H3 lysine 9 (H3K9) and downregulation of Notch1 expression, thereby inhibiting inflammation. Ultimately, NaHS improves depressive behavior in type 1 diabetes mice by modulating the inflammatory response and the ferroptosis signaling pathway and their correlation (WANG et al., 2021b).

Ketamine (non-competitive antagonist of N-methyl-D-aspartate (NMDA) receptors) is a racemic compound consisting of equal amounts of R-ketamine and Esketamine. Esketamine has demonstrated clear antidepressant effects, and its nasal spray has been approved by the United States and Europe as an adjunctive therapy to oral antidepressants for treatment-resistant depression (TRD) in adults (Jelen et al., 2021). Studies have suggested that the antidepressant properties of ketamine may be associated with downstream mechanisms involved in regulating synaptic plasticity, including brain-derived neurotrophic factor (BDNF), eukaryotic elongation factor 2 kinase (eEF2K), mechanistic target of rapamycin (mTOR), and glycogen synthase kinase-3 (GSK-3) (ZANOS and GOULD, 2018). Zhang et al. conducted a study using a CRS rat model and found that ketamine can rapidly exert antidepressant effects by inhibiting the ferroptosis signaling pathway within nuclear complexes. Transmission electron microscopy revealed that CRS rats exhibited increased expression of TfR1 and decreased expression of FTH1 and GPX4, leading to ferroptosis, mitochondrial contraction, and chromatin condensation. Treatment with ketamine reversed these pathological reactions, leading to an improvement in depressive-like behavior in CRS rats (Zhang et al., 2022).

**FIGURE 3:**
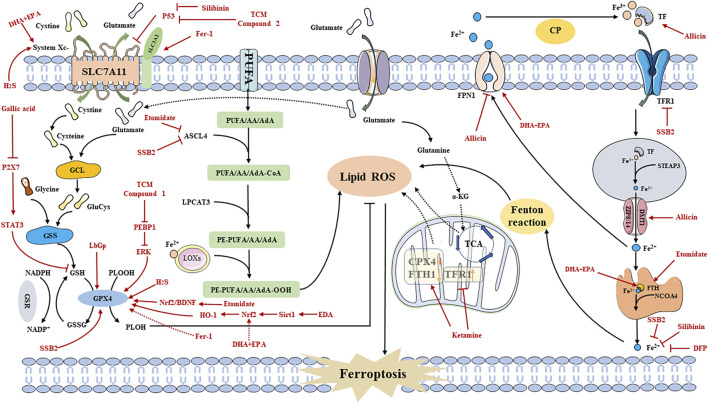
Regulating ferroptosis to Treat Depression Mechanisms. Fer-1: ferrostatin-1; DHA: docosahexaenoic acid; EPA: eicosapentaenoic acid; SSB2: Saikosaponin B2; DFP: Deferiprone; TCM: traditional chinese medicine; PEBP1: Phosphatidylethanolamine Binding Protein 1; EDA: Edaravone; Sirt1: silent mating-type information regulation 2 homolog 1; HO-1: Heme oxygenase-1; Nrf2: Nuclear Factor erythroid 2-Related Factor 2; BDNF: brain-derived neurotrophic factor; EPK: extracellular regulated protein kinases; LbGp: Lycium barbarum glycopeptide; STAT3: signal transducers and activator of transcription 3; H_2_S: Hydrogen sulfide; CP: Ceruloplasmin.

Electroconvulsive therapy (ECT) is an effective method for treating severe depression (REN et al., 2014). This procedure involves intravenous anesthesia, neuromuscular block, and mechanical ventilation. Commonly used intravenous anesthesia agents include propofol and etomidate, with etomidate having been shown to be more effective than propofol in terms of ECT efficacy (GUREL et al., 2022). Li et al. demonstrated, using a CUMS rat model, that etomidate used in ECT can protect hippocampal neurons from ferroptosis by modulating the BDNF/Nrf2 pathway, thereby enhancing the antidepressant effect of ECT. Nrf2 is an important player in the ferroptosis signaling pathway. The use of etomidate upregulates the expression of BDNF, Nrf2 and GPX4, thereby inhibiting ferroptosis in hippocampal neurons and improving depression-like states in rats. To further investigate the role of ferroptosis in ECT, the researchers administered ferrostatin-1 (Fer-1), as the most potential ferroptosis inhibitor (LONG et al., 2023), to the rats. Western blot analysis indicated significant upregulation of SLC7A11 and GPX4 in the CUMS model rats injected with Fer-1, while the levels of free Fe^2+^ decreased. Moreover, the combination of etomidate in ECT and Fer-1 exhibited a stronger antidepressant effect compared to etomidate in ECT alone (Li et al., 2023). Moreover, Li et al. discovered that Fer-1 reversed the upregulation of tsRNA-3029b induced by CUMS in mice. tsRNA-3029b knockdown suppressed ferroptosis and promotes cell regeneration of Corticosterone (CORT)-induced neuronal cells, leading to an improvement in depressive-like behavior in mice (LI E. et al., 2023). These findings demonstrate that both etomidate in ECT and Fer-1 reversed depressive behavior and hippocampal neuronal loss (Figure 3; Table 3).

**TABLE 3:** Regulation of antidepressant chemicals on ferroptosis.

Antidepressant chemical	Chemical structure	Molecular formula	CAS number	Moulding method	Animal or cell type	Dosage of drugs used	Behavioral testing evaluation	Antidepressant mechanisms	References
Deferiprone	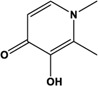	C_7_H_9_NO_2_	30,652-11-0	5-HTT KO	C57BL/6Jmice	50 mg/kg/d	PST, Locomotor Activity, NSFT	May potentially modulate unstable iron pools in the brain with amelioration of oxidative stress, thereby improving depressive-like behavior	UZUNGIL et al. (2022)
Edaravone	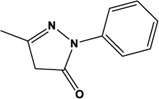	C_10_H_10_N_2_O	89-25-8	CSDS	C57BL/6J mice、CD-1 mice	10 mg/kg/d	SPT, OPT, EPMT, TST, FST, NORT	Improvement of Depression-like Behavior through Modulation of the Sirt1/Nrf2/HO-1/Gpx4 Signaling Pathway	DANG et al. (2022)
Eicosapentaenoic acid	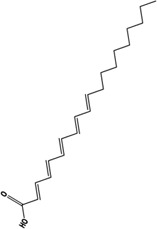	C_20_H_30_O_2_	32,839-30-8	PTZ	ICR mice	1%,w/w	OFT, FST, TST	Improvement of depression by reversing the expression of transferrin proteins such as TfR1, FPN1 and FTH1 and inhibiting ferroptosis through modulation of the Nrf2/HO-1/System Xc-/GSH/GPX4 signaling pathway	Wang et al. (2022)
Docosahexaenoic acid	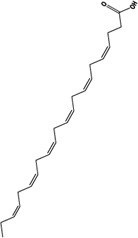	C_22_H_32_O_2_	6217-54-5	PTZ	ICR mice	1%,w/w	OFT, FST, TST	Inhibition of ferroptosis and inflammation ameliorates depression by reversing the expression of transferrin proteins such as TfR1, FPN1, and FTH1 and modulating the Nrf2/HO-1/System Xc-/GSH/GPX4 signaling pathway	Wang et al. (2022)
H_2_S	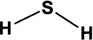	H_2_S	7783-06-4	STZ	C57BL/6J mice	5.6 mg/kg/d	OFT, EPMT, FST, TST	Improvement of depression through modulation of System Xc-/GSH/GPX4 signaling pathway in ferroptosis, inhibition of pro-inflammatory cytokine release and enhancement of sirtuin 6 (Sirt6) protein expression	WANG et al. (2021b)
Ketamine	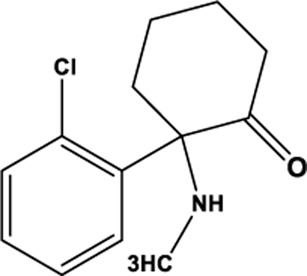	C_13_H_16_ClNO	100,477-72-3	CRS	Wistar-Kyoto (WKY) rats	10 mg/kg	OFT, SPT, FST, TST	Inhibition of ferroptosis by reversing TfR1, FTH1 and GPX4 expression ameliorates depression	Zhang et al. (2022)
Etomidate	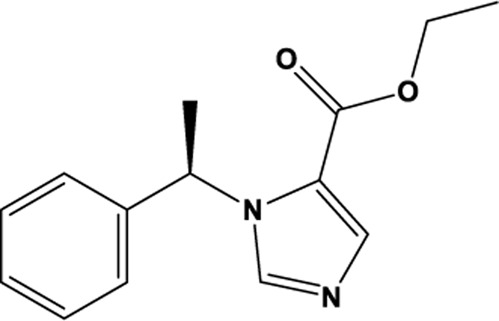	C_14_H_16_N_2_O_2_	33,125-97-2	CUMS	Sprague-Dawley rats	20 mg/kg	OFT, SPT, FST	Inhibition of ferroptosis through modulation of GSH/GPX4 signaling pathway, lipid metabolism pathway, and expression of transferrin FTH in ferroptosis ameliorates depression	Li et al. (2023)
Ferrostatin-1	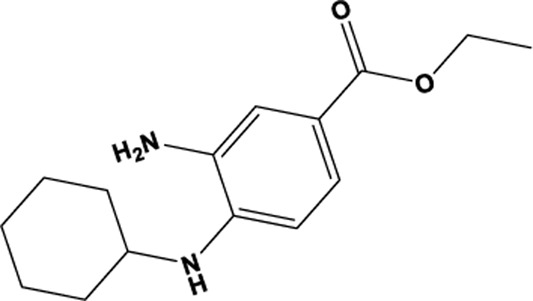	C_15_H_22_N_2_O_2_	347,174-05-4	CUMS	Sprague-Dawley rats	2 mg/kg	OFT, SPT, FST	Inhibition of ferroptosis through modulation of System Xc-/GSH/GPX4 signaling pathway in ferroptosis ameliorates depression	Li et al. (2023)
				CORT, CUMS	C57BL/6 mice	5 mg/kg	SPT, OPT, TST, FST	Inhibition of ferroptosis and promotion of CORT-induced cell regeneration in neuronal cells by downregulation of tsRNA-3029b ameliorates depression	LONG et al. (2023)

5-HTT KO: serotonin transporter knockout; CUMS: chronic unpredictable mild stress; CRS: chronic restraint stress; CSDS: chronic social defeat stress; CRS: chronic restraint stress; CORT: corticosterone; STZ: streptozotocin; PTZ: pentylenetetrazole; OFT: open field test; TST: tail suspension test; FST: forced swimming test; SPT: sucrose preference test; EPMT: elevated plus maze test; NSFT: novelty-suppressed feeding test; NORT: novel object recognition test; Nrf2: nuclear factor (erythroid-derived 2)-like 2; SIRT1: silent mating-type information regulation 2 homolog 1; GSH: glutathione; GPX4: glutathione peroxidase 4; System Xc-: cystine/glutamate transporter; HO-1:Heme oxygenase-1; TfR1: transferrin receptor 1; FTH1: Ferritin Heavy Chain 1; FPN1: ferroportin 1.

In summary, abnormal ferroptosis signaling pathways leading to reduced neurotransmitter synthesis, lipid peroxide accumulation, mitochondrial dysfunction, and increased release of pro-inflammatory factors are important factors contributing to decreased neural plasticity, delayed synaptic growth and development, impaired neural network conduction, and neurotoxicity, ultimately leading to depression. The aforementioned studies provide compelling evidence for the critical role of ferroptosis in the pathogenesis and treatment of depression.

## 6 Conclusion and outlook

Depression is a prevalent mental disorder characterized by persistent symptoms such as low mood and reduced interest, with severe cases sometimes leading to suicidal tendencies, and has been a major contributor to poor global health and disability, with a recently increasing incidence (ZHANG X. et al., 2022; SHAHBAZI et al., 2022; SU and SI, 2022; YAO et al., 2022). Despite the continuous development of antidepressants, the treatment options for depression remain limited, often failing to provide sufficient relief for patients (WANG L. et al., 2023). Therefore, there is an urgent need to explore new strategies for developing antidepressant drugs (LEVENBERG and CORDNER, 2022). Recent research progress on the mechanism of ferroptosis suggests a close association between ferroptosis dysregulation and the pathological mechanism of depression (MAES et al., 1995b; WANG et al., 2019; LIANG et al., 2020; BERTHOU et al., 2021; WANG L. et al., 2023). This paper explored the efficacy of TCM ingredients, compound formulas, and antidepressant chemicals in regulating ferroptosis to combat depression, based on a thorough review of relevant literature reports.

While active ingredients and compound prescriptions of TCM hold significant research value, several challenges need to be addressed. The current focus on regulating the ferroptosis signaling pathway for depression treatment is primarily limited to animal models, lacking clinical research to validate its efficacy in patients. Many active ingredients in TCM present issues like poor stability, solubility, and difficulty crossing the BBB, which restrict their use in treating depression effectively. Furthermore, existing experimental research primarily targets single pathways or signals, neglecting the interactions between different targets or pathways. Additionally, validation methods such as gene knockout and antagonists are lacking, warranting further evidence to support the specificity of TCM and its targets. While antidepressant chemicals have shown positive effects in preclinical studies, their clinical application is limited due to side effects such as hallucinations, hepatotoxicity, neurotoxicity, and addiction. Similarly, ECT may result in adverse reactions like cardiac complications, cognitive impairment, and apnea, significantly limiting its use in clinical settings.

In future research, it is crucial to collect additional preclinical and clinical evidence, specifically focusing on the unique advantages of TCM in treating depression. It is necessary to conduct multicenter, high-quality, double-blind randomized controlled trials to further explore whether TCM formulas and their active ingredients can effectively regulate the ferroptosis signaling pathway, thereby improving depressive symptoms. Additionally, there is a need to enhance research on targeted delivery systems for active ingredients to increase drug concentration in the CNS and improve treatment efficacy (GERAILI et al., 2021). Integration of multiple omics techniques should be employed to explore additional ferroptosis signaling pathways and targets for depression treatment and prevention. It is also important to utilize reverse validation methods such as blockers or gene knockouts to further elucidate the mechanisms involved in the targeted regulation of ferroptosis by TCM formulas and active ingredients. Considerable work remains in exploring the regulatory mechanisms of antidepressant therapies on ferroptosis, both in clinical and preclinical studies. These efforts are of great significance for the advancement of antidepressant research and development.
